# Enrichment and Quantification of Epitope-specific CD4+ T Lymphocytes using Ferromagnetic Iron-gold and Nickel Nanowires

**DOI:** 10.1038/s41598-018-33910-0

**Published:** 2018-10-24

**Authors:** Daniel E. Shore, Thamotharampillai Dileepan, Jaime F. Modiano, Marc K. Jenkins, Bethanie J. H. Stadler

**Affiliations:** 1 Chemical Engineering & Materials Science, University of Minnesota, Minneapolis, MN USA; 2 Department of Microbiology and Immunology, University of Minnesota, Minneapolis, MN USA; 3 Animal Cancer Care and Research Program, University of Minnesota, St. Paul, MN USA; 4 Veterinary Clinical Sciences (College of Veterinary Medicine), University of Minnesota, Saint Paul, MN USA; 5 Masonic Cancer Center, University of Minnesota, Minneapolis, MN USA; 6 Center for Immunology, University of Minnesota, Minneapolis, MN USA; 7 Electrical and Computer Engineering, University of Minnesota, Minneapolis, MN USA

## Abstract

Epitope-specific CD4+ T lymphocytes were magnetically enriched using ferromagnetic Ni and Fe-Au nanowires coated with a monomer containing a major histocompatibility complex class II-bound peptide epitope (pMHCII). The enriched lymphocytes were subsequently quantified using fluorescence-activated cell sorting (FACS). This was the first use of magnetic nanowires for cell sorting using FACS, and improvements in both specificity and fluorescent signal strength were predicted due to higher particle moments and lengths than conventional paramagnetic beads. Three different types of nanowires (Ni, Fe with Au tip and Fe-Au multilayers) were made by electrodeposition. Ni nanowires separated fewer T cells than Au tipped Fe nanowires, likely because Ni has a lower magnetic moment than Fe. Fe-Au multilayer nanowires separated more T cells than Au-tipped Fe nanowires because there was more monomer per nanowire. Also, increasing the amount of monomer increased the number of CD4+ cells separated. Compared to conventional paramagnetic beads, the nanowires had lower specificity for CD4+ T cells, but had stronger fluorescent signals due to more fluorophores per particle. This results in broader FACS baseline separation between the positive and negative cells, which is useful to detect T cells, even those with lower binding affinity for pMHCII ligands.

## Introduction

**Figure 1:**
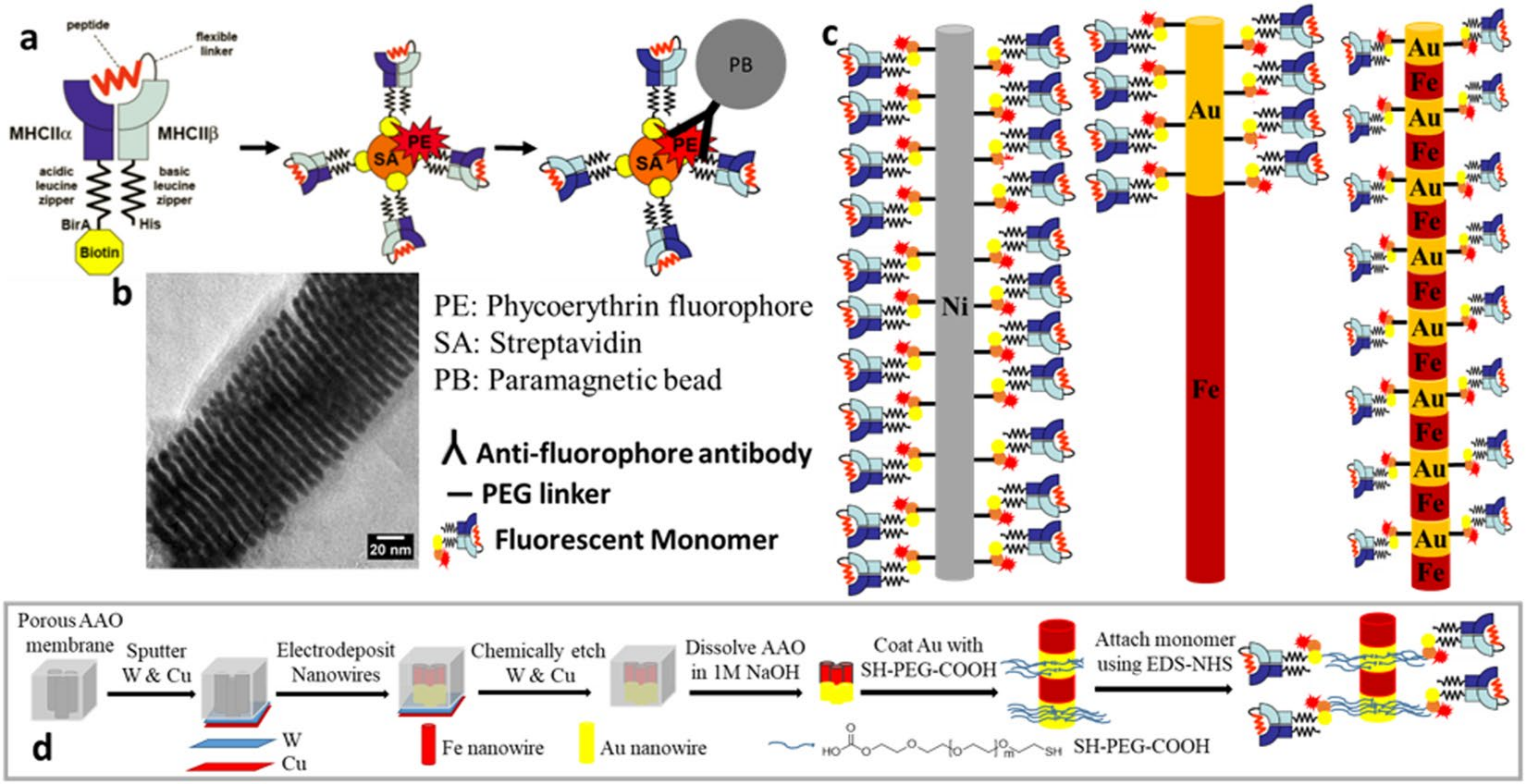
(**a**) Cartoon of monomer structure and fluorescently-tagged tetramer with paramagnetic bead (PB) attached to the tetramer using an antibody. (**b**) TEM image of Fe-Au multilayer nanowire, darker layers are Au, lighter layers are Fe. (**c**) Cartoon diagrams (not to scale) showing fluorescently-tagged nanowire multimers, Ni on the left, Au-tipped Fe in the middle and Fe-Au multilayer on the right. (**d**) Multi-step process for nanowire fabrication, coating with SH-PEG-COOH, and attachment of fluorescently-tagged monomer.

Immunologists are interested in understanding how CD4+ T lymphocytes regulate the immune response. These lymphocytes use T cell antigen receptors (TCRs) to recognize microbial peptides bound to major histocompatibility complex class II (MHCII) molecules on infected host cells. Because each T cell has a different TCR, only a few cells can recognize any given MHCII-bound peptide epitope (pMHCII) and thus only a few can respond during a specific infection. Detecting rare cells has been challenging. One approach involves pMHCII tetramers that are formed by decorating fluorescent streptavidin molecules with four biotin-labeled pMHCII monomers^1^ (Fig. 1a). These fluorescent tetramers can be used to tag, track, and quantify populations of epitope-specific T cells using fluorescence-activated cell sorting (FACS). Some groups have attached biotinylated pHMC monomers to fluorescent streptavidin molecules using a sub-stoichiometric ratio^2,3^. Biotinylated dextran was then used to create multimers with more than four monomers to tag and quantify T cells using FACS^2,3^.

To use the tetramers for magnetic cell separation and T cell quantification, commercial paramagnetic beads (embedded in a plastic, spherical particle) are attached to the fluorescent tetramer using an antibody that binds to the fluorescent streptavidin molecules. Then, the specific T cell population can be magnetically enriched before FACS. There are no published reports that use dextramers for magnetic cell separation. Here, we investigate using ferromagnetic nanowires in place of the paramagnetic beads in an attempt to improve cell enrichment efficiency and fluorescent quantification accuracy.

Many studies have found that ferromagnetic nanowires are comparable or more effective at cell enrichment than paramagnetic beads^4–13^. For example, Hultgren *et al*. found that bare Ni nanowires (350 nm in diameter, 35 µm long) captured up to 75% of NIH-3T3 mouse fibroblast cells, compared to 35% for iron oxide paramagnetic beads (diameter 1–2 µm)^4^, and cell enrichment was optimized when the nanowire length (5–35 µm) matched the diameter of the target cells^5,12^. Gao *et al*. coated 25 µm Ni nanowires with antibodies to label mouse endothelial cells (MS1) and found the optimal nanowire concentration for cell enrichment was 2.0 × 10^6^ ml^−1^, due to increased nanowire aggregation at higher concentrations^6^. These previous studies did not use FACS, so their nanowires were much longer than those used in the present work, and they used cells from a cell culture with one cell type. Here, a mixture of T-lymphocyte cells harvested from the spleens of C57BL/6 (B6) mice was used. Kim *et al*. used antibody functionalized Ni-silicide (NiSi) nanowires (60–100 nm diameter, 5–10 µm long) to tag and separate CD4+ T lymphocyte cells from a mixture of lymphocyte cells. They found ~95% capture efficiency for both the nanowires and the commercial paramagnetic beads^13^. Multiple groups have shown that electrodeposited Ni nanowires have minimal cytotoxicity^5,6^ especially if the surfaces are coated with PEG and/or RGD^11,14^.

Ferromagnetic nanowires are made by electrodeposition of either single metals or multilayered metals (Fig. 1b) into insulating templates that contain columnar nanopores. Nanowire length can be controlled (from hundreds of nm to tens of microns) using time and/or charge counting during deposition. If nanowires and nanowire-labelled cells are to be sorted with a flow cytometer after tagging (as in our work), then the length should be limited to avoid clogging or damage. The nanowires in this current study were less than 3 µm long, whereas the previous studies used nanowires greater than 5 µm long.

The materials chosen for our study are high saturation magnetization ferromagnetic metals (415 emu/cc for Ni^4^ and 1685 emu/cc for Fe^15^) compared to commercial iron oxide paramagnetic beads (25–40 emu/cc^4,16^). Increased magnetization will increase the magnetic forces during enrichment. In this work, we used Ni, Au-tipped Fe, and multilayered Fe-Au nanowires to create fluorescent “multimers,” with multiple monomers attached to each nanowire as compared to four monomers per tetramer. The Au layers were added to the nanowires because the Au segments are easily coated with SH-PEG-COOH^17^. This helps mitigate nanowire aggregation, increases biocompatibility^14^, and acts as a flexible linker to attach the fluorescent streptavidin and monomers to the nanowires (Fig. 1c). The length, and therefore surface area, of the Au segments can be easily adjusted during electrodeposition of the nanowires. A larger Au surface area allows many fluorescent pMHCII monomers to attach to each nanowire (Fig. 1c). Having more fluorophores per particle should increase the strength of the fluorescent signal detected by FACS to improve detection of T cells even when TCRs have a low affinity for the pMHCII ligand^2,3^. All these factors make electrodeposited nanowires an attractive alternative to paramagnetic beads for magnetic cell enrichment.

## Results

### Characterization of nanowires and monomer functionalization

**Figure 2:**
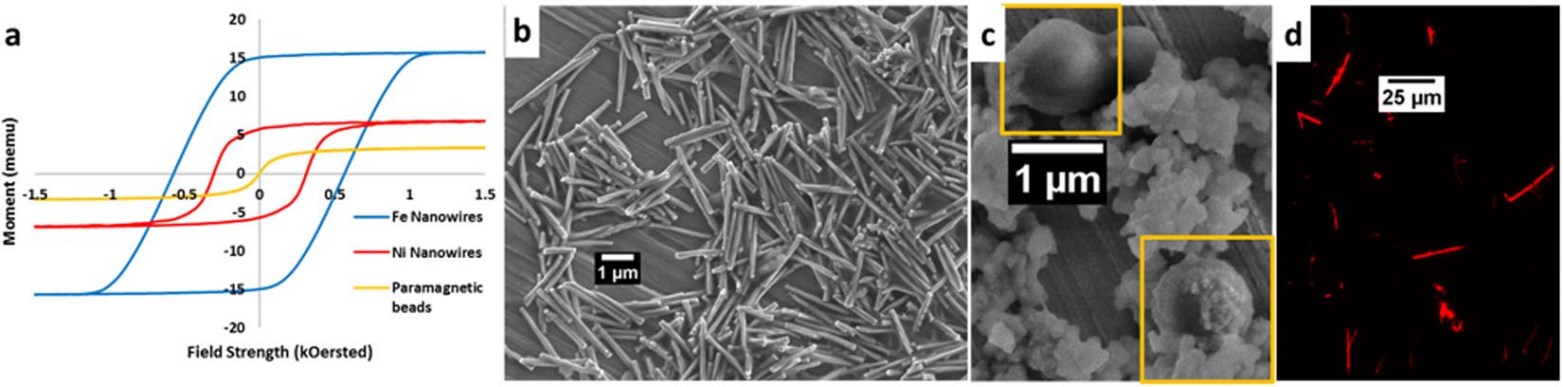
(**a**) Hysteresis curves for Fe nanowires (blue), Ni nanowires (red), and paramagnetic iron oxide beads (yellow). (**b**) SEM image of ferromagnetic Ni nanowires. (**c**) SEM image of paramagnetic beads; note the debris around the beads is likely a coating (**d**). Fluorescent microscopy images of Ni nanowires coated with fluorescently-tagged monomers, note these nanowires were not used for cell enrichment experiments.

Following previous successful cell enrichment studies, Ni nanowires were used to make our first fluorescent multimers^4–6^. We then compared the performance of Ni nanowires to Fe-Au nanowires to study the effect of increased magnetization, Fig. 2a, since Ni has a specific magnetization 415 emu/cc compared to 1685 emu/cc for Fe. To the best of our knowledge, this is the first work using electrodeposited Fe-Au nanowires for cell enrichment.

All the Ni and Fe-Au nanowires were 100 ± 12 nm in diameter. In the first study, comparing Ni to Fe nanowires, the Ni nanowires (Fig. 2b) were 2.94 ± 0.08 µm long while the Au-Fe nanowires (Fig. 1b) were 1.92 ± 0.28 µm with 125–130 nm of Au on one end. In the second study, comparing Fe-Au nanowires to paramagnetic beads, the Au-tipped Fe nanowires were 0.85 ± 0.1 µm long, the Fe-Au multilayer nanowires were 0.85 ± 0.24 µm and 1.43 ± 0.36 µm, and the paramagnetic beads were ~1 µm in diameter (Fig. 2c). A fluorescent, biotinylated pMHCII (2W:I-A^b^) monomer was attached to the Ni nanowires using a NH_2_-PEG-COOH linker (MW 1000), and for the Fe-Au nanowires a flexible SH-PEG-COOH linker (MW 1000) was covalently bonded to the Au surfaces. In both cases, a fluorescent streptavidin was then attached by an EDC-NHS reaction to link the –COOH group (Fig. 2a). Lastly, the monomer was attached to the streptavidin on the nanowires via biotin linkage. The fluorescent monomer attachment was confirmed using fluorescent microscopy, Fig. 2d.

### Comparing Ni and Au tipped Fe nanowires for cell enrichment

**Figure 3:**
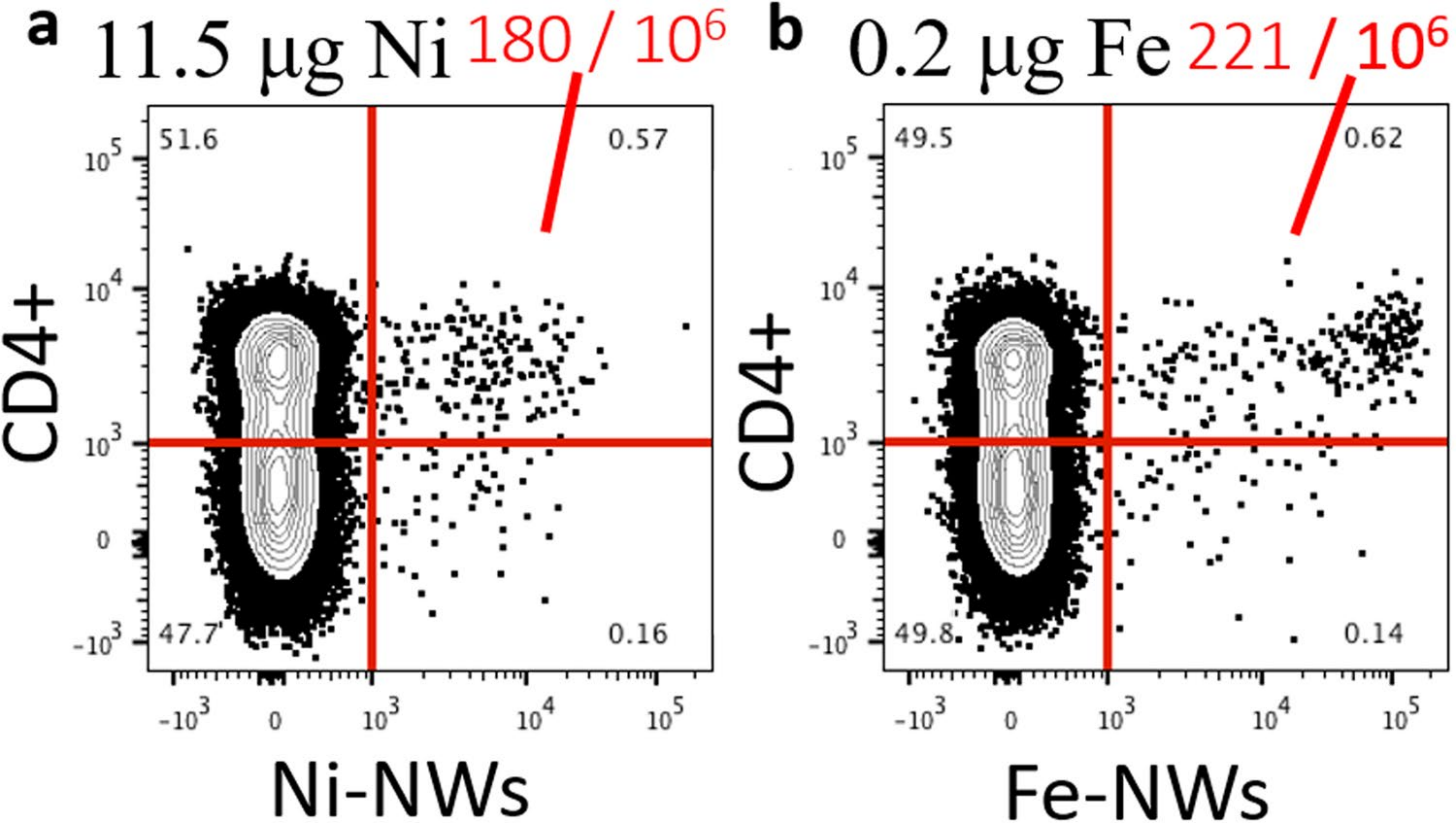
Flow cytometric analysis of CD4+ T lymphocytes cell suspensions after tagging and magnetic enrichment with fluorescent Ni (left) or Fe (right) nanowire conjugates and magnetic enrichment.

Three different doses of Ni nanowires and Fe-Au nanowires were prepared with the same amount of 2W:I-A^b^ monomer for each dose. The different nanowires were mixed with single cell suspensions and incubated with spleen and lymph node cells from 2W-immune mice for 30 minutes at 4 °C in the dark before magnetic enrichment. After magnetic enrichment, the cells were washed to remove untagged cells and re-suspended to fluorescently stain the cell membranes with lineage-specific antibodies for quantification of the fluorescent nanowire bound cells using flow cytometry. In the cytometry plots (Fig. 3), the target cells were marked with Alexa Fluor 700-conjugated CD44 (expressed by antigen-experienced T cells) antibody, and APC fluorophore labeled 2W:I-A^b^-nanowires. The number of double positive CD4+ T cells were identified in the top right quadrant of each plot. The thresholds for this quadrant were determined by comparing the fluorescent signals from CD4+ cells extracted from an immunized mouse versus CD4 cells extracted from a non-immunized mouse. There are distinct populations in the FACS plots for the CD4+ cells from the immunized mouse based on the cell size and light scattering properties. The results in Fig. 3 show that the Fe-Au nanowires detected more target T cells than the Ni nanowires for much less mass of Fe (0.2 µg) compared to Ni (11.5 µg). The results for two lower doses are shown in Supplementary Fig. S1. Each batch of nanowires had the same amount of monomer, and the Fe nanowires (1.9 µm) were shorter than the Ni nanowires (2.9 µm), therefore the improved cell separation is likely due to the higher magnetization of the Fe nanowires.

In these plots, the target cells of interest bound both Alexa Fluor 700-labeled CD44 antibody and 2W:I-A^b^-APC fluorophore labeled nanowires. This means that the nanowire concentrations were low enough that magnetic aggregation did not impede cell enrichment, as was observed in previous cell enrichment work with much longer Ni nanowires^6^.

To improve quantification of different cell types using flow cytometry immunologists want a wider baseline, which is the signal separation between the target and non-target cells on the x-axis of the cytometry plot. The Fe-Au nanowires had a wide baseline between the double positive cells (Fig. 3, top right quadrant) and the negative cells in two left side quadrants. Wider baselines make it less likely to mistakenly count a positive cell as a negative cell.

### Comparing Au tipped Fe, and Fe-Au multilayer nanowires with paramagnetic beads

**Figure 4:**
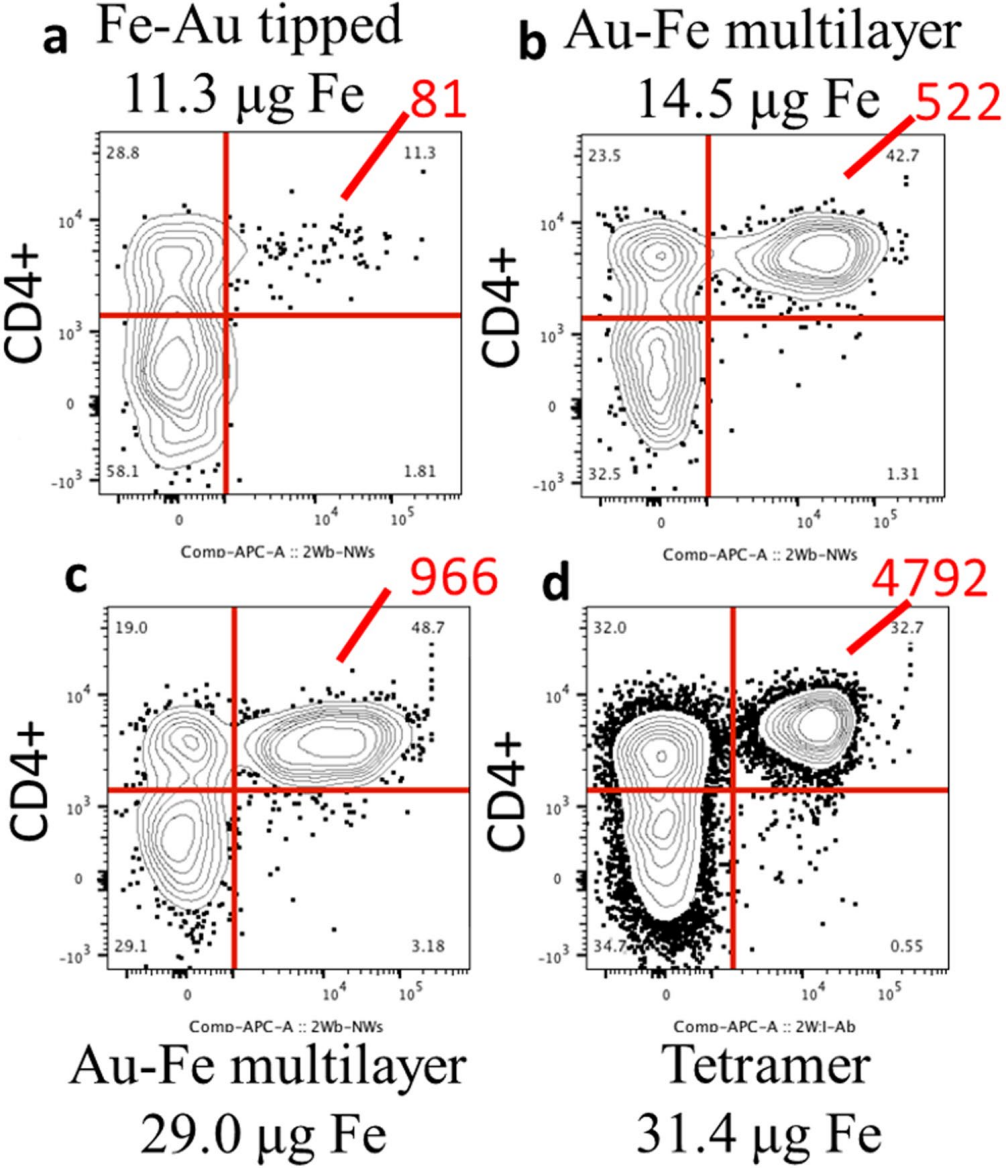
Flow cytometric analysis of CD4+ T lymphocytes cell suspensions after tagging and magnetic enrichment with fluorescent Au-tipped Fe nanowires (**a**) or Fe-Au multilayer nanowires (**b**,**c**) or conventional tetramer (**d**).

After determining that Fe-Au nanowires were more effective than Ni nanowires for cell enrichment, the Fe-Au nanowires were used for comparing these nanowire-based multimers with conventional tetramers. Two different types of Fe-Au nanowires were prepared: one type with Au tips on the Fe nanowires and another with alternating Fe and Au layers down the length of the nanowires, Fig. 1c. The multilayer structure allows fluorescent pMHCII monomer to coat the entire length of the nanowire (similar to the Ni nanowire multimers in the previous section) instead of only the tips of the nanowires. Figure 4a,b show that the Fe-Au multilayer 2W:I-A^b^-nanowires separated more than 5 times as many cells as the Au-tipped Fe nanowires (522 cells versus 81 cells) using a similar mass of Fe (14.5 µg versus 11.3 µg Fe).

Figure 4c,d show the cytometry results for a larger dose of Fe-Au multilayer nanowires, 29 µg Fe, with a similar dose of iron oxide paramagnetic beads, 31.4 µg Fe, for comparison. The multilayer 2W:I-A^b^ nanowires separated 20% as many cells as the 2W:I-A^b^ tetramer, most likely because the nanowires may have detached from the T cells during enrichment due to torque or drag forces on the high magnetic moment nanowires. It is also possible that some of the nanowires with T cells remained stuck to the inside of the polypropylene microcentrifuge tubes after magnetic enrichment because of the PEG coating and the larger magnetic force pulling the nanowires to the side. Kim *et al*. also enriched CD4+ T cells with NiSi nanowires, but they used antibodies to bind the nanowires to T cells instead of pMHCII monomers. They observed only slightly lower enrichment efficiency compared to paramagnetic beads (93.5% versus 96.8%). The pMHCII monomer binding is orders of magnitude weaker than the antibody binding, and magnetic moment for the NiSi nanowires is lower than for Fe-Au nanowires^13^; both of these factors may explain why they enriched a higher percentage of CD4+ T cells. Kim *et al*. also quantified the percent of cells separated differently; rather than counting the cells tagged with nanowires in FACS, they used FACS to count the percent of CD4+ T cells in suspension before magnetic enrichment and then after magnetic enrichment they counted the unseparated cells remaining in the wash.

Importantly, the fluorescent signal from the Fe-Au 2W:I-A^b^ nanowires had a broader baseline than the fluorescent signal from the 2W:I-A^b^ tetramer. In fact, many cells had signals at ~10^5^ for the fluorophore. The stronger signal from the nanowires is likely due to increased monomer coating each particle.

By systematically increasing the mass of Fe-Au multilayer nanowires (14.5, 29, and 87 µg Fe) and keeping monomer loading constant, the number of cells separated increased, from 522, 966, and 1506 cells separated (Supplementary Fig. S2). However, the highest Fe-Au dose only separated 31% as many CD4+ T cells as the tetramers (Supplementary Fig. S2). Another experiment used 163 µg of Fe-Au multilayer nanowires and separated 28% as many cells as the tetramers. This suggests that increasing the nanowire mass can improve cell separation, but only up to a certain point. There may be nanowire aggregation at higher masses, as observed in a previous study by Gao *et al*.^6^. It is also likely that increasing the nanowire mass increased the number of non-specific lymphocyte cells tagged and separated by the nanowires. In a similar experiment Fe-Au multilayer 2W:I-A^b^ nanowires (length: 1.43 ± 0.36 µm, mass: 32.5 µg) were used to tag and separate CD4+ T cells and compared with the 2W:I-A^b^ tetramer. Unfortunately, the nanowires tagged and separated many more non-target CD8+ T cells than the tetramer (Supplementary Fig. S3, bottom row), likely because each nanowire had a much larger surface area for non-target cell attachment. This agrees with the results from Hultgren *et al*. that nanowires are more efficient than paramagnetic beads for non-specific cell enrichment^4^.

To verify that increased monomer loading increases the number of cells separated, three identical Fe-Au multilayer nanowire samples were prepared (2.5 ± 0.28 µm long, 163 μg of Fe in each) and coated with 0.02, 0.1 and 0.2 nanomoles of streptavidin and monomer molecules (ratios 1:5:10, respectively). After tagging and magnetic enrichment with these 3 samples and a tetramer control sample, and the 1X, 5X, and 10X samples tagged and separated 81, 557, 1143 CD4+ T cells, respectively (Supplementary Fig. S4). The tetramers separated 4127 CD4+ T cells, so the Fe-Au multimer samples separated 2%, 13.5% and 27.7% as many cells as the tetramers. These percentages are approximately proportional to the ratios of monomer used in each sample. This indicates that the increased CD4+ Tagging separation was likely due to the increased monomer loaded on the nanowires.

Again, the nanowires had a broader baseline than the tetramers, with many cells having signals at ~10^5^ for the APC fluorophore. The signal intensity from these nanowire-based multimers was also higher than that reported for dextran based dextramers^2^, but there are no published reports of dextramers with magnetic particles for comparison of magnetic cell enrichment.

## Discussion

Ferromagnetic Ni and Fe-Au nanowires were fabricated by electrodeposition and used to label CD4+ T cells for magnetic enrichment and fluorescent quantification using FACS. Au-tipped Fe nanowires separated more cells than Ni nanowires, using the same amount of monomers but less magnetic mass, likely because Fe has a larger magnetic moment than Ni. Fe-Au multilayered nanowires had the best enrichment, followed by Au-tipped Fe, and Ni nanowires. Fe-Au multilayer nanowires separated more CD4+ T cells than Au tipped Fe nanowires likely because the pMHCII monomer coated the length of the nanowire, so the cells could attach to down the length of the nanowire. The number of CD4+ cells was approximately proportional to the amount of monomers used to prepare the nanowires, for identical Fe-Au multilayer nanowire samples. Although the Fe-Au multilayer and Au tipped nanowires detected fewer specific CD4+ T cells than the conventional tetramer, they had a broader baseline separation between the positive and negative cells, with many cells having signals at ~10^5^ for the APC fluorophore. This feature may be useful for detection of T cells that bind to their pMHCII ligand with relatively low affinity.

## Methods

### Materials

Nanoporous anodic aluminum oxide (AAO) (1 cm^2^) with pore diameters 100 ± 12 nm were obtained from Synkera Technologies, Inc. A gold cyanide plating solution HS434 RTU was obtained from Technic Inc. Heterobifunctional polyethylene glycol NH_2_-PEG-COOH (M.W 1000) was obtained from Creative PEGWorks. Heterobifunctional polyethylene glycol (PEG), SH-PEG-COOH, (M.W 1000) and 2-(N-morpholino)ethanesulfonic acid (MES buffer) were obtained from Sigma Aldrich. 1-Ethyl-3-[3-dimethylaminopropyl]carbodiimide hydrochloride (EDC) and N-hydroxysulfosuccinimide (Sulfo-NHS) were purchased from Thermo Scientific. A two position magnetic stand (1.5 ml MagnaSphere Technology, face field of 0.1 T and field gradient of 20 T/m) from Promega was used for nanowire and cell enrichment.

### Functionalization of nanowires

The nanowires were fabricated by a well-known process using electrodeposition into AAO templates; see the supplementary information for a detailed discussion of nanowire fabrication. The nanowires were 0.84–2.94 µm long and 100 ± 12 nm in diameter For the Ni nanowire based multimers the Ni nanowires were first coated with NH_2_-PEG-COOH with the –COOH groups sticking out from the nanowires to couple with streptavidin. An aqueous solution of NH_2_-PEG-COOH, 0.2% weight, in 0.5 M NaCl was made and the pH was adjusted to 12–12.5 using 0.1 M NaOH. The Ni nanowires were resuspended in 1 ml of this NH_2_-PEG-COOH overnight at room temperature. For the Fe based multimers the Fe-Au nanowires were resuspended in 1 mL of 1 mM SH-PEG-COOH aqueous solution overnight to attach the –SH group to the Au surfaces. For both the Ni and Fe-Au nanowires the PEG solution was aspirated and the nanowires were rinsed with deoionized (DI) water to remove any unbound PEG. The PEG nanowires were then resuspended in 1 ml of pH 6.0 MES buffer to use the EDC-NHS reaction to attach the streptavidin fluorophores to the nanowires. The EDC-NHS reaction was done according to the instructions from Thermo Scientific. EDC was added to the nanowires in MES to a concentration of 10 mM (10-fold excess of –COOH) followed by NHS to a concentration of 25 mM (2.5-fold EDC) and reacted for 15 minutes at room temperature. Next, the MES buffer was aspirated and the nanowires were rinsed 1X in pH 7.0 PBSA (phosphate buffered saline with 0.01% Sodium Azide) to remove any free EDC and NHS and raise the pH. Then the nanowires were resuspended in 100 µL of PBSA solution and 100 µL of 14 µM fluorescent streptavidin allophycocyanin (APC) or Phycoerythrin (PE) fluorophore at room temperature for 2 hours to bond the streptavidin to the nanowires via an amide bond. Afterwards the streptavidin was aspirated and nanowires washed with PBSA 3 times to remove any unbound streptavidin. A biotin-labeled 2 W peptide:MHCII (I-A^b^) monomer solution, prepared as described by Moon *et al*.^1^, was added to the nanowires, with a final monomer concentration of 200 µg/ml (3 µM), the concentration was diluted with PBSA. The nanowires were stored in the dark for 30 min at 4 °C to bind the monomer to the streptavidin on the nanowires via a biotin linkage. Unbound monomer was aspirated and washed away with PBSA 3 times. The fluorescent nanowire multimers were stored in 500–800 µl of PBSA in the dark at 4 °C until aliquots were added to the single cell suspension for tagging and enrichment. At this point 20 µl was removed for each nanowire or tetramer sample to determine the Fe or Ni concentration. The Ni or Fe concentration was quantified by adding 20 µl of concentrated HNO_3_ and leaving overnight to dissolve the nanoparticles to Ni^2+^ or Fe^3+^ ions respectively. A Bruker Minispec mq60 NMR Analyzer at 1.5 T (60 MHz) was used to measure the *T*_1_ relaxation time, which is highly sensitive to Ni^2+^ or Fe^3+^ ion concentrations, for each sample and these were compared with a calibration curve of known concentrations of NiCl_2_ or FeCl_3_ to calculate the Ni or Fe mass used in each cell enrichment^17^.

### Preparing paramagnetic bead-based tetramer

The tetramer solution was prepared by first mixing 25 µL of 2W:I-A^b^ monomer solution (monomer in PBSA a concentration 1.3 mg/ml) with 25 µL of streptavidin-PE (10 µl – Prozyme [streptavidin] = 8.8 uM, [APC] = 6.5 µM) for 30 min at 25 °C and then overnight at 4 °C. Then 50 µL iron oxide based Miltenyi paramagnetic beads were added to the tetramer solution for 30 min at 4 °C so the anti-APC antibody could bind to the APC fluorophore on the tetramer.

### Raising and immunizing mice

4- to 8-week old C57BL/6 (B6) mice were purchased from the Jackson Laboratory or the National Cancer Institute Mouse Repository. Mice were bred and housed under specific pathogen–free conditions according to the guidelines of the University of Minnesota Institutional Animal Care and Use Committee (UMNIACUC) and the National Institutes of Health. All experiments were approved by the UMNIACUC and were performed in accordance with relevant guidelines and regulations of this committee. Mice were used at 6–12 weeks of age. Mice were given subcutaneous injection of 100 μl (split over two sites) of complete Freund’s adjuvant emulsion (Sigma-Aldrich) containing 100 μg of 2 W peptide (GenScript) in Dulbecco’s PBS (Life Technologies)^1^.

### CD4+ T cell tagging and magnetic enrichment and staining

The T cells were harvested from 2 W peptide-immunized C57BL/6 (B6) mice. Single cells suspensions were prepared by removing the spleen and lymph nodes from the B6 mice, mechanically mashing the organs, and filtering out debris^1^. For each experiment, the cell suspension was divided into 250 µL samples for each nanowire and tetramer sample, so each sample should have a similar number and concentration of CD4+ cells and other background cells. Nanowire samples ranging from 50–300 µL were added to each cell suspension in a 2 ml conical tube; for the tetramer sample 2 µL of the tetramer solution (from a 1 µM stock), final tetramer concentration of 10 nM, was added to the cell suspension. After addition of the different nanoparticle solutions, the cell suspensions were put in the dark for 1 hour at 4 °C for cell tagging. The tubes were gently mixed at a 45° angle during tagging. After tagging the cell suspensions were transferred to 1.7 ml centrifuge tubes for magnetic enrichment. Each tube was placed in magnetic stand that applied a face field of 0.1 T and field gradient of 20 T/m for 5 min for cell enrichment. Each sample was washed 3 times with 1 ml PBSA to rinse away unbound cells and then bound cells resuspended in PBSA for cell staining.

### Fluorescence-activated cell sorting (FACS) and analysis

After cell enrichment and washing, the cells were stained with fluorochrome-labeled antibodies specific for informative cell surface markers, dump channel (B220, CD11c, CD11b), CD3, CD4, CD8 and CD44 (as described by Moon *et al*.)^1^. After cell staining, each sample was resuspended in ~400 µL of FACS buffer for flow cytometry on a Fortessa (BD). Data were analyzed using FlowJo software (TreeStar).

## Electronic supplementary material

Dataset 1
