# Supplementary material for: Enrichment and Quantification of Epitope-specific CD4+ T Lymphocytes using Ferromagnetic Iron-gold and Nickel Nanowires
Source: Sci Rep. 2018 Oct 24;8:15696. doi: 10.1038/s41598-018-33910-0 (PMC6200781; doi:10.1038/s41598-018-33910-0)
Supplement: Supplementary file 1 — Dataset 1 [file 41598_2018_33910_MOESM1_ESM.pdf]

Supplementary Information

Enrichment and Quantification of Epitope-specific CD4+ T Lymphocytes using Ferromagnetic Iron-gold and Nickel Nanowires

Daniel E. Shore[1,+], Thamotharampillai Dileepan[2,+], Jaime F. Modiano[3,4,5,6], Marc K. Jenkins[2], & Bethanie J. H. Stadler[1,4,*]

[1]Chemical Engineering & Materials Science, University of Minnesota, Minneapolis, MN 55455.

[2]Lab Medicine and Pathology (School of Medicine), University of Minnesota, Minneapolis, MN 55455.

[3]Animal Cancer Care and Research Program, University of Minnesota, St. Paul, MN.

[4]Veterinary Clinical Sciences (College of Veterinary Medicine), University of Minnesota, Saint Paul, MN 55108.

[5]Masonic Cancer Center, University of Minnesota, Minneapolis, MN.

[6]Center for Immunology, University of Minnesota, Minneapolis, MN.

[7]Electrical and Computer Engineering, University of Minnesota, Minneapolis, MN.

*Email: stadler@umn.edu

[+] These authors contributed equally to this work.

Nanowire Fabrication

The multistep process for nanowire fabrication and coating is shown in Fig. 1d in the main text. The anodic aluminum oxide (AAO) membranes were sputter-coated on one side with 20 nm W and 300 nm Cu for electrical contact. After sealing the contact side of the AAO, the nanowires were electrodeposited inside the membranes using a platinum mesh counter electrode (Technic Inc.) and a Ag/AgCl glass electrode (BASi) the reference electrode. For the Au tipped Ni and Fe nanowires the HS434 RTU Au cyanide plating solution was used to deposit an Au layer at constant current of -100 µA for 5 min at 40°C. For the Ni nanowires the electrolyte had 0.4 M H_3BO_3, 0.3 M NH_4Cl (adjusted to pH 3 with 1 M NaOH) 0.001 M malonic acid and 0.02 M $NiSO_4$; the Fe electrolyte (for Fe tipped nanowires) had 0.02 M $FeSO_4$ instead of Ni SO_4 and the Fe-Au electrolyte had an additional 0.002 M $KAu(CN)_2$. For the Ni nanowires, Ni was deposited at constant voltage of -0.9V from the $NiSO_4$ bath at room temperature. For the Fe-Au nanowires pulsed deposition was used (to improve nanowire uniformity) with one pulse at -1.1V for 10 secs (for Fe) and -50 µA (for rest, or Au layer) for hundreds of pulses. The Cu-W growth electrode was removed by first dissolving the Cu layer in 1 M $FeNO_3$ for 1 min, then dissolving the W layer in 30% H_2O_2 for 1 min. The AAO membrane was etched in 5% weight NaOH for 2 hours. An ultrasonicator bath set at 33 kHz and 1 Hz sweep was used to ultrasonicate the AAO, once every 20 minutes for 2 minutes to free the nanowires into solution. Using a magnetic stand, the nanowires were collected at the walls of 1.7 ml the plastic centrifuge tubes washed 3 times with DI water.

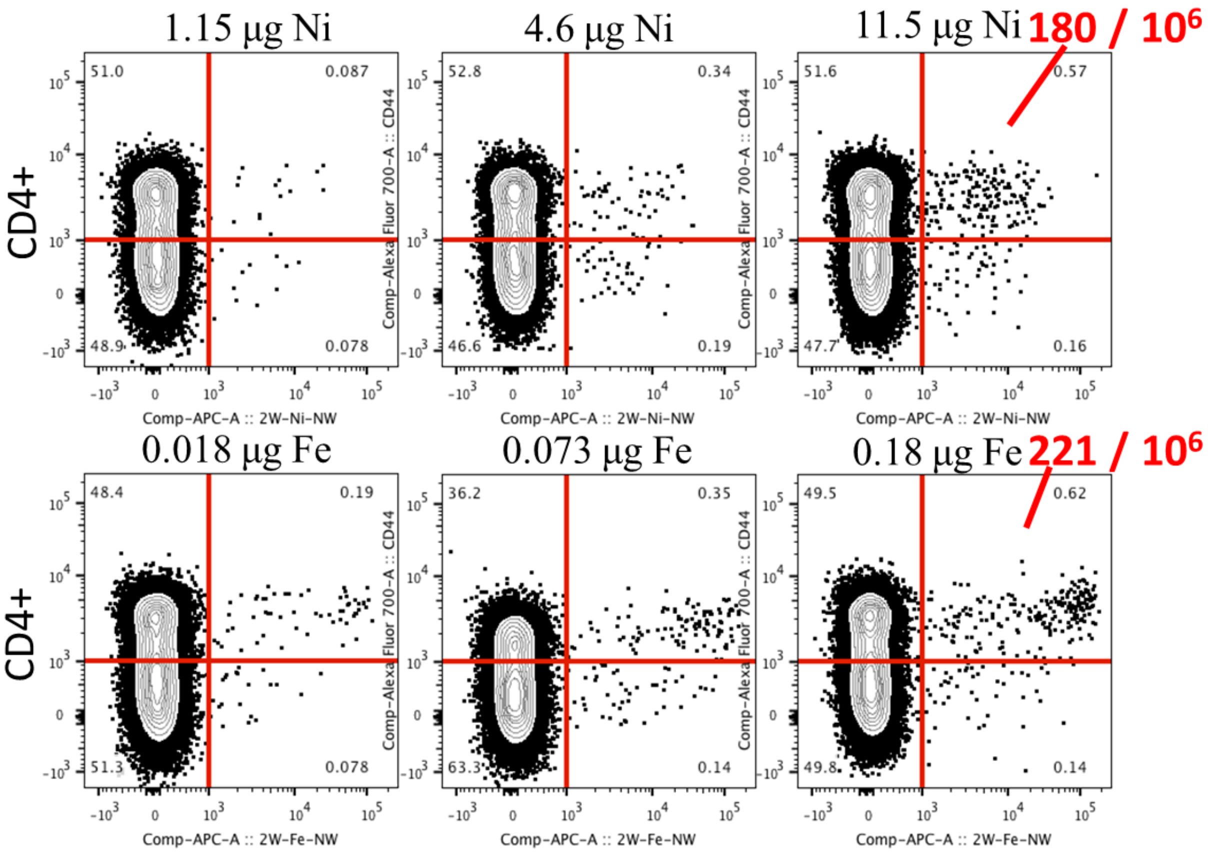

Supplementary Figure S1. Flow cytometric analysis of CD4+ T lymphocytes cell suspensions after tagging and magnetic separation with 3 doses of Ni (top row) or Fe (bottom row) fluorescent nanowire multimers.

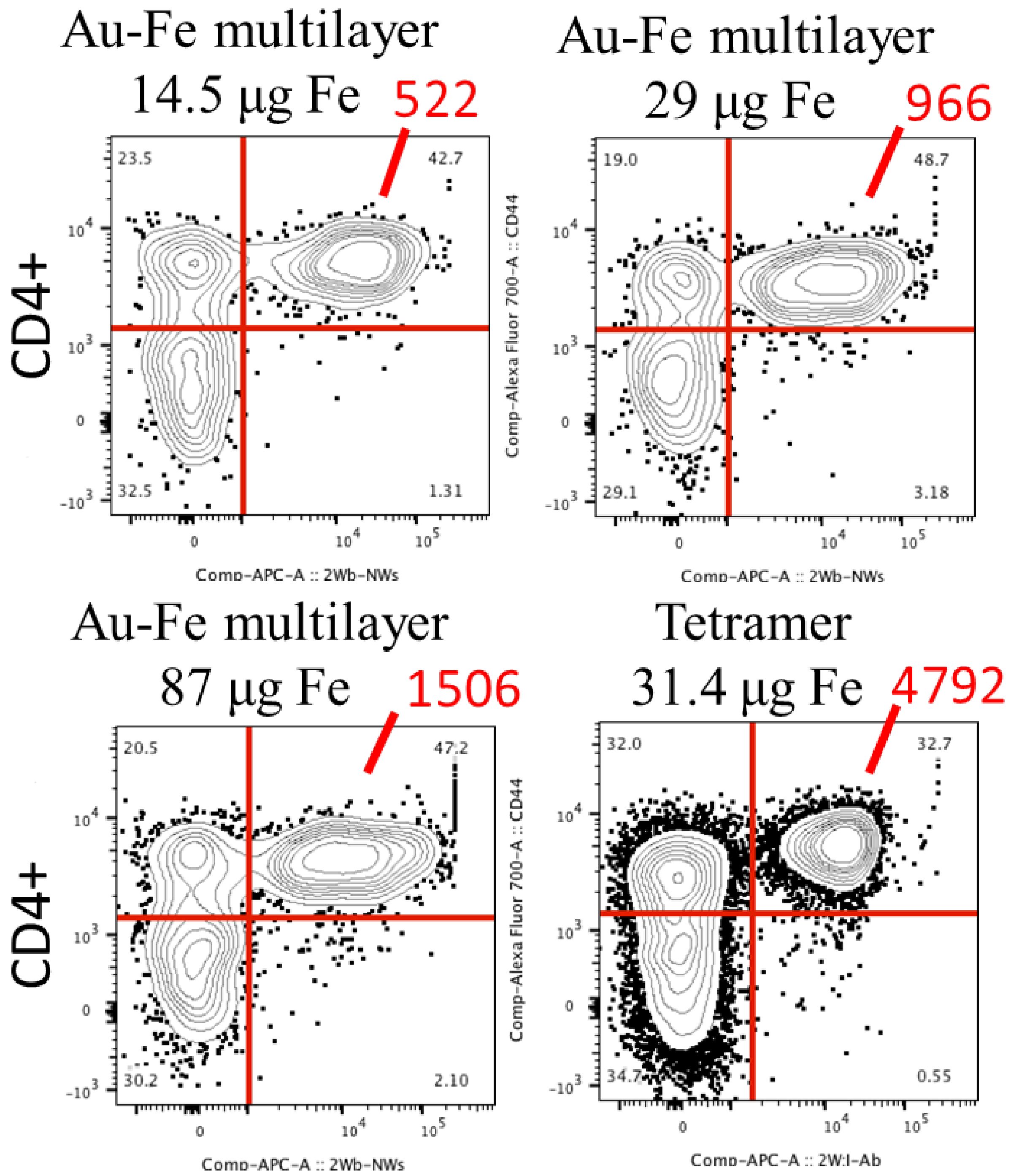

Supplementary Figure S2. Flow cytometric analysis of CD4+ T lymphocyte cell suspensions after tagging and magnetic separations with 3 different doses of Fe-Au multilayer fluorescent multimers. The cytometric results of another separation with fluorescent tetramers is also shown for comparison. Note that the top right and two bottom plots were shown in figure 4 of the main text.

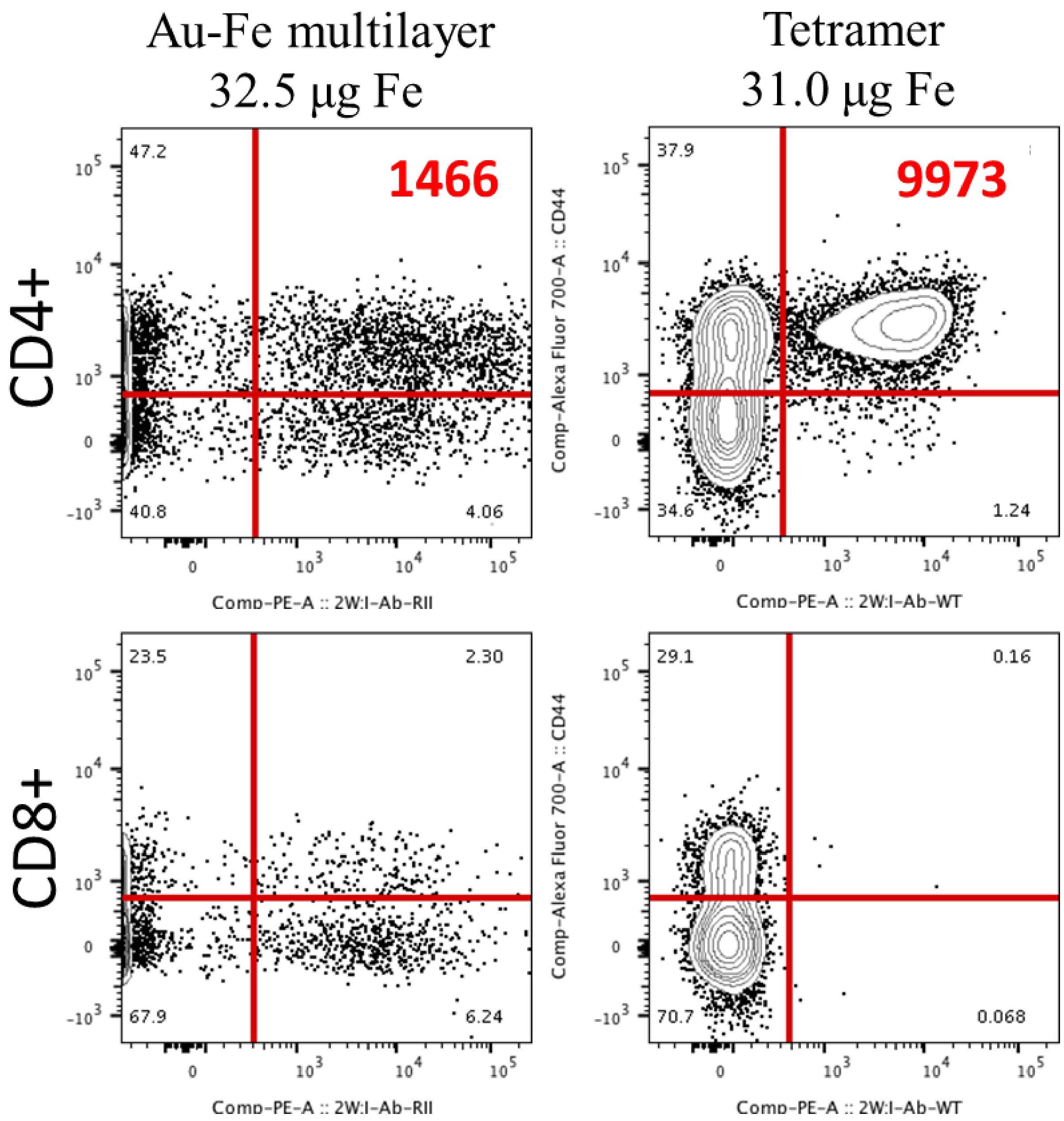

Supplementary Figure S3. Flow cytometric analysis of CD4+ and CD8+ T lymphocyte cell suspensions after tagging and magnetic separations with Fe-Au multilayer fluorescent multimers (top left), length 1.43±0.36 µm, and fluorescent tetramers. The bottom row shows the results for the CD8+ T cell counts for the same multimer and tetramer separations.

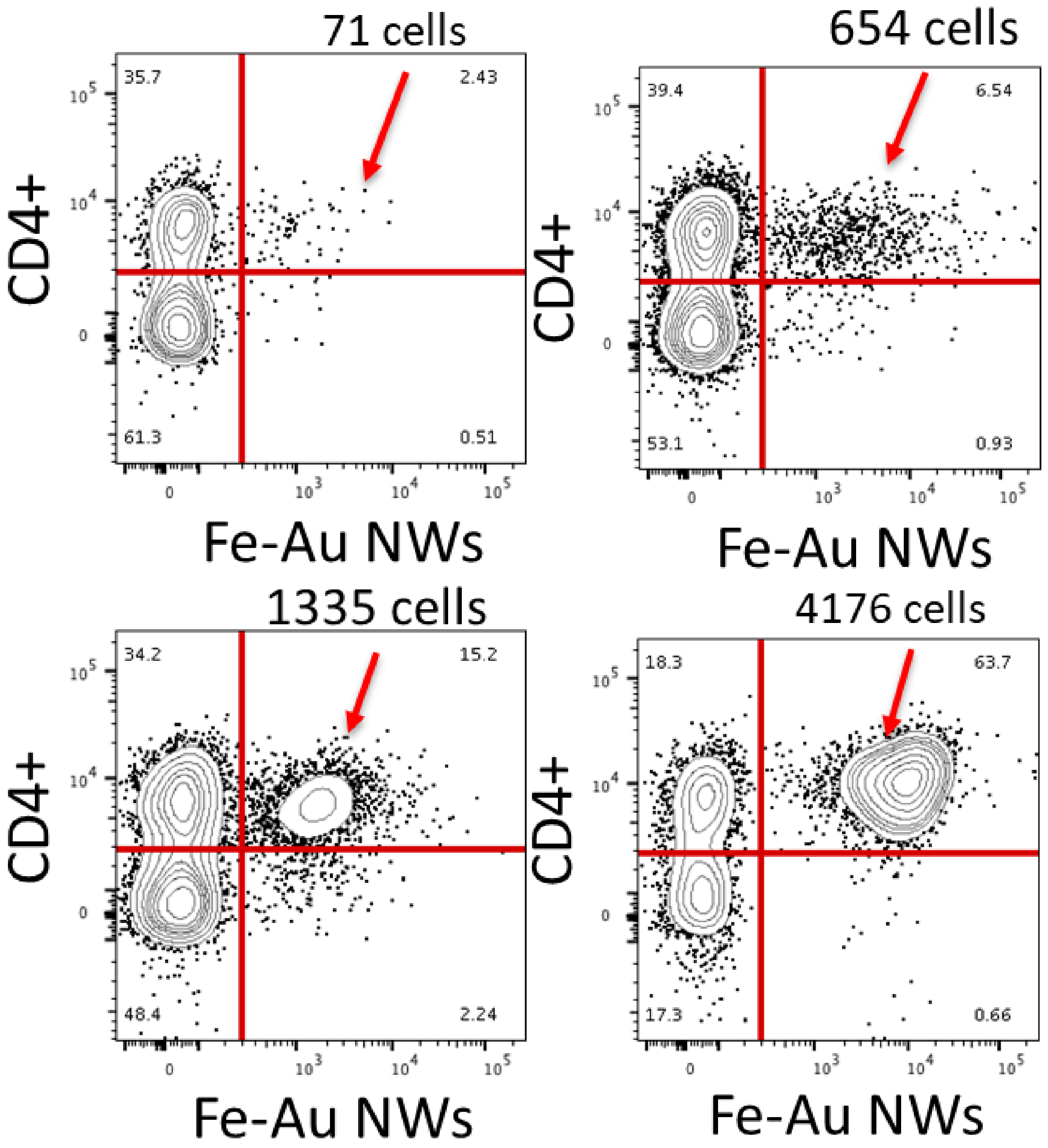

Supplementary Figure S4. Flow cytometric analysis of CD4+ T lymphocyte cell suspensions after tagging and magnetic separations with Fe-Au multilayer fluorescent multimers and tetramers. Top left, nanowires loaded with 0.02 nanomoles of monomer. Top right, nanowires loaded with 0.1 nanomoles of monomer. Bottom left, nanowires loaded with 0.2 nanomoles of monomer. Bottom right, tetramers used for cell separation.
